# Transcriptome-based exon capture enables highly cost-effective comparative genomic data collection at moderate evolutionary scales

**DOI:** 10.1186/1471-2164-13-403

**Published:** 2012-08-17

**Authors:** Ke Bi, Dan Vanderpool, Sonal Singhal, Tyler Linderoth, Craig Moritz, Jeffrey M Good

**Affiliations:** 1 Museum of Vertebrate Zoology, University of California, Berkeley, 3101 Valley Life Sciences Building, Berkeley, CA, 94720-3160, USA; 2 Division of Biological Sciences, University of Montana, Missoula, MT, 59812, USA; 3 Department of Integrative Biology, University of California, Berkeley, 1005 Valley Life Sciences Building, Berkeley, CA, 94720-3140, USA

**Keywords:** Microarray-based exon capture, Phylogenetics, Population genomics, SNP identification, *Tamias*, Target enrichment

## Abstract

**Background:**

To date, exon capture has largely been restricted to species with fully sequenced genomes, which has precluded its application to lineages that lack high quality genomic resources. We developed a novel strategy for designing array-based exon capture in chipmunks (*Tamias*) based on *de novo* transcriptome assemblies. We evaluated the performance of our approach across specimens from four chipmunk species.

**Results:**

We selectively targeted 11,975 exons (~4 Mb) on custom capture arrays, and enriched over 99% of the targets in all libraries. The percentage of aligned reads was highly consistent (24.4-29.1%) across all specimens, including in multiplexing up to 20 barcoded individuals on a single array. Base coverage among specimens and within targets in each species library was uniform, and the performance of targets among independent exon captures was highly reproducible. There was no decrease in coverage among chipmunk species, which showed up to 1.5% sequence divergence in coding regions. We did observe a decline in capture performance of a subset of targets designed from a much more divergent ground squirrel genome (30 My), however, over 90% of the targets were also recovered. Final assemblies yielded over ten thousand orthologous loci (~3.6 Mb) with thousands of fixed and polymorphic SNPs among species identified.

**Conclusions:**

Our study demonstrates the potential of a transcriptome-enabled, multiplexed, exon capture method to create thousands of informative markers for population genomic and phylogenetic studies in non-model species across the tree of life.

## Background

High-throughput, next generation sequencing (NGS) technologies and associated bioinformatics tools have fundamentally changed the scale at which DNA sequence data can be gathered and analyzed [1]. NGS allows for a massive amount of sequence data to be affordably and quickly obtained. In principle, these approaches can be implemented without prior genomic knowledge of the focus species, thus offering tremendous potential for addressing various novel and long-standing evolutionary questions previously hampered by technology and cost [2].

NGS allows researchers to investigate genome-wide molecular, structural, and regulatory mechanisms underlying adaptation, diversification, and speciation [3]. NGS also enables comparative genome scans for polymorphism which can then be used to infer demography and selection [4]. Molecular phylogenetics also benefits from the increasing accessibility of NGS. Large-scale, multi-locus data (i.e., hundreds to thousands of loci) combined with improved analytical tools for inferring gene trees, provides unprecedented opportunities for resolving species phylogenies [5]. Toward this end, a core challenge of population genomic and phylogenetic studies is obtaining a reliable set of orthologous loci from a sufficient number of individuals across populations or species spanning a range of divergences [6]. Even though the cost of NGS continues to fall, most evolutionary labs cannot sequence whole genomes or a large portion of genomic regions from samples spanning divergent clades. Moreover, whole genome data simply is not necessary to answer many research questions. In this context, genome partitioning and targeted re-sequencing of a consistent subset of genomic regions will remain the most cost-effective and analytically straightforward approach for most evolutionary applications. Genome partitioning with targeted DNA capture allows for the selective NGS of thousands of genomic regions [7], facilitating rapid assays of genetic variation. Compared to partitioning methods that search for anonymous markers (i.e. restriction site associated DNA tags, or RADtags [8], DNA capture is expected to be more efficient for finding orthologous markers among divergent genomes [6,9,10]. When applied to exonic regions, DNA capture can also provide information on gene function and evolution. Exon capture involves the hybridization of genomic libraries to short oligonucleotide baits complementary to complete or partial exomes printed on a microarray [7] or attached to magnetic beads in solution [11]. The captured exon-containing DNA fragments of individual or pooled genomic libraries are then eluted from the array and the target-enriched elute is sequenced using an NGS platform. To date, the design of exon capture relies heavily on existing high quality genomic resources (e.g. [12]). However, the genomes of most organisms of ecological and evolutionary interest are yet to be sequenced, which has largely impeded the expansion of DNA capture across the tree of life.

**Figure 1:**
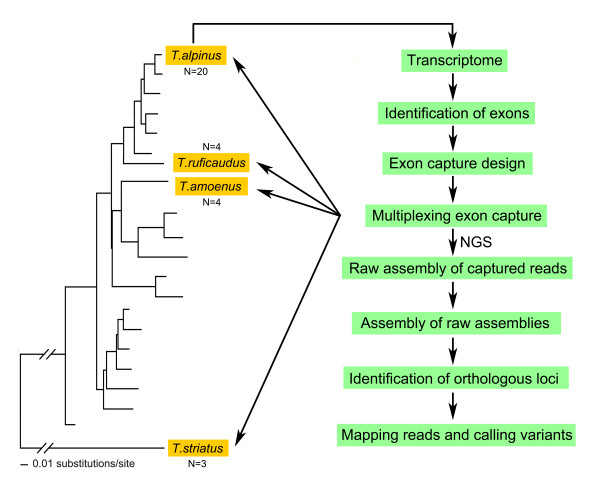
**An overall work flow of this study.** The *Tamias* phylogenetic tree is modified from [13] by replacing the outgroup species with *T.striatus*. The *Tamias* species that were not under investigation in the present study are not shown.

In this study, we propose a series of methods (Figure 1) aimed at adapting exon capture based NGS to organisms without pre-existing reference genomes. Here we focused on array-based capture but note that the same general principles should directly extend to an in-solution approach. We focused on North American chipmunks of the genus *Tamias* to test our methods. *Tamias* are the focus of a comprehensive set of studies that aim to understand their evolutionary history, patterns of hybridization, and gene introgression (e.g., [13,14]). There is no reference genome currently available for this group; at the onset of our study the most closely related genomic resource was a low-coverage (2X) draft genome of the thirteen-lined ground squirrel (*Ictidomys tridecemlineatus*), which is around 30 million years (My) divergent from *Tamias*. The house mouse (*Mus musculus*) and rat (*Rattus norvegicus*) are the closest high-quality reference genomes, but last shared a common ancestor with chipmunks around 70 My. In this context, we developed genomic resources by first sequencing multi-tissue transcriptomes from one chipmunk species (the alpine chipmunk, *Tamias alpinus*), and then designed arrays by targeting a subset of exons from the annotated transcripts. Furthermore, to test how increased divergence affects capture efficiency, we included anonymous genomic targets from the thirteen-lined ground squirrel on this array. We then tested the feasibility of this approach by using these arrays to capture sequence from four chipmunk species, spanning the range of genetic divergence in this genus. Up to 20 individually indexed genomic libraries from each species were pooled and hybridized on single arrays, enabling highly cost-effective sequencing ( Additional file 1) on a scale that is desirable for diverse population genomic and phylogenetic applications. Finally, we developed novel methods for analyzing exon capture data in the absence of a reference genome. 

## Results and discussion

### *Tamias alpinus* transcriptome

The *T. alpinus* multi-tissue cDNA library generated 30,233,530 raw sequence reads with a total length of 3.02 Gb (NCBI SRA ID: SRA053502). After filtering, the total length of cleaned reads was 2.69 Gb. The *de novo* assembly information is summarized in Additional file 2. Instead of subjectively selecting a single kmer size for transcriptome assembly, we explored multiple-kmer assemblies followed by merging raw assemblies and removing redundancies, as recommended by previous studies [15-17]. Previous work has demonstrated that a multiple k-mer strategy increases both contig length and transcript diversity when compared to a single k-mer assembly method [15]. The mean contig length among the 20 raw assemblies generated by ABySS ranged from 771 to 1,074 bp with an overall average of 902 bp. The final merged consensus assemblies contained 37,563 contigs (36.5 Mb) with a mean length of 972 bp. Among these contigs, 21,994 (28.1 Mb) showed strong orthology (BLASTX E-values < 1e-10) with known proteins from the combined human (*Homo sapiens*), mouse, rat and thirteen-lined ground squirrel protein dataset. The annotated transcripts matched 11,320 (49%) of the unique protein-coding genes in the Ensembl mouse protein database. Among these contigs, 55.5% covered the full coding sequences of a gene including partial sequences of 5’ and 3’ untranslated regions (UTRs), 26.2% partially covered coding sequences with partial 3’ UTR, 8.1% covered partial coding sequences with partial 5’ UTR, and 10.2% only contained partial coding sequences. As expected, the annotated contigs are 3’ UTR biased because the mRNA were enriched through oligo-dT selection [18]. The mean length of the annotated transcripts was 1,297 bp and the average base coverage was 54X (median 11X). There was a positive relationship between transcript length and the number of reads mapped, but we found no strong correlation between coverage and the length of contigs ( Additional file 3: Figure S1). Errors during the transcriptome assembly appear to be trivial. The percentage of open reading frames (ORFs) that contained premature stop codons in the annotated contigs, which could be derived either from pseudogenes or by assembly errors, was only 1%. We also found that 1.2% of the annotated contigs comprised more than one distinct transcript, which were spuriously combined during the assembly. The array probes were designed according to the exons identified by comparing mouse protein to mouse genomic DNA, not the entire transcriptome. Thus, the low level of chimeria present in our dataset is unlikely to introduce errors during the array design. We were able to identify 127,456 putative exons across 21,262 annotated transcripts.

### Exon capture array design

We used all identified exons that were longer than 200 bp (average size 332 bp) in length to design tiling probes for the capture arrays. Note that shorter targets (≥ 60 bp) are possible, but targeted capture performance is expected to decline for relatively short regions. In addition to the *T. alpinus* exons, we also targeted regions from the *Tamias* mitochondrial genome, 162 anonymous *I. tridecemlineatus* genomic intervals, the Y-linked SRY gene, and seven previously sequenced nuclear genes (See M&M for details). After filtering probes that likely contained repetitive elements, 962,438 probes were synthesized onto the Agilent 1M arrays.

Probe design is critical for successfully enriching orthologous loci with variable rates of evolution from cross-species DNA hybridization. Ideally, probes should reflect the full range of evolutionary rates of loci. While highly conserved genes are expected to provide more efficient capture across species, they will often be less useful for resolving phylogenetic relationships. At the other extreme, rapidly evolving loci will reduce the efficiency of cross-species hybridization. Furthermore, they might tend to reflect signatures of natural selection to a greater extent, and are thus more likely to introduce bias into the inference of phylogenetic relationships among species. Our goal was to design an array that targeted the full genomic range of evolutionary rates in an unbiased manner. We compared *T. alpinus* annotated transcripts against the transcriptome of Belding’s ground squirrel (*Urocitellus beldingi*), which was sequenced and assembled with the same method described above (data not shown). We found that the average sequence divergence between all identified orthologous regions from the two species was 5.2 ± 3.4%. The divergence between the targeted *T. alpinus* exons and the corresponding *U.beldingi* orthologs was 5.0 ± 2.9%, and followed a distribution similar to that of the overall divergence between the two species ( Additional file 3: Figure S2). These results indicate that our array design is unlikely to be biased towards highly conserved genic regions.

### Exon capture data filtration and assemblies

The libraries for all individuals within a single species were pooled into a species library. Four exon capture arrays were used to capture each of the species libraries. After capture, the *T. alpinus* library (n=20) was sequenced on one lane, and the *T. amoenus* (n=4), *T. ruficaudus* (n=4)*,* and *T. striatus* (n=3) libraries were equally combined and sequenced on two lanes. Summary information for the de-multiplexed libraries is shown in Additional file 4. In total, 11.2 and 29.1 Gb of raw sequence data were respectively obtained for the *T. alpinus* (SRA053501) species library and for the combined library of *T. amoenus* (SRA053503), *T. ruficaudus* (SRA053504), and *T. striatus* (SRA053505) species libraries. The sequence quality among individual libraries on each lane was consistent. For example, across the 20 *T. alpinus* libraries, the overall mean base quality score was 33.7 ± 0.1, while 84.7 ± 0.3% of the bases in the raw sequence reads had quality scores greater than 30.

The results for data filtration are shown in Additional file 5. The raw reads of each individual library were filtered to remove exact duplicates, adapters, bacteria and human contamination, as well as low complexity and quality reads. On average, 90.7% of the raw *T. alpinus* reads, and 71.2-72.6% of the raw reads for the other *Tamias* species passed all of these filters. Compared to the percentage of read duplicates present in *T. alpinus* libraries (1.5%), there was a substantial portion of raw reads identified as duplicates in *T. amoenus* (20.7%), *T. ruficaudus* (19.8%), and *T. striatus* (20%). Increased data yield makes repeated sequencing of the same molecule, and thus duplicates, more likely in PCR amplified libraries [19]. These results indicate that additional sequencing of the captured *T. alpinus* libraries would result in higher unique coverage, while additional sequencing effort in the other species would present increasingly diminishing returns. Duplicate reads were removed in all subsequent analyses to avoid inappropriate pseudo-replication.

The cleaned reads of each species library assembled by ABySS and SOAP*denovo* produced 24 and 25 raw assemblies, respectively ( Additional file 6). The mean and median lengths, and N50 of assemblies generated by various combinations of k-mer and k-cov were similar among species ( Additional file 3: Figure S3). The raw assemblies were merged by species to produce consensus assemblies, which were then compared to the *T. alpinus* exons and other targeted regions to identify the portion of consensus assemblies that are associated with various targets (in-target assemblies). As expected, the length of a contig in the assemblies was much greater than the length of the corresponding targeted *T. alpinus* exon ( Additional file 7), illustrating the potential for exon capture to obtain sequence information at flanking intronic regions even when probes are restricted to exons.

### Capture efficiency

**Figure 2:**
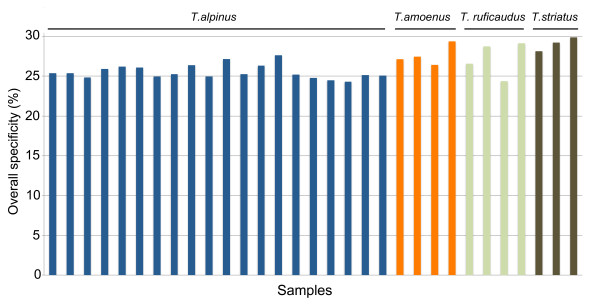
**Specificity of exon capture in the four chipmunk species.** Specificity denotes the percentage of cleaned reads aligned within the targets over the total reads aligned to the consensus assemblies. The number of individual libraries barcoded and pooled on the same array varies for different species, while the overall specificity among the 31 specimens is similar. Each column represents an individual library.

We retrieved the complete *Tamias* mitochondrial genome and all 7 nuclear control loci from all 31 individual libraries. For the targeted *T. alpinus* exons and for all libraries, the sensitivity, or percentage of exons that were covered by at least one read, was greater than 99%, which is consistent with other exome capture studies (e.g. [7,20]). For the 20 *T. alpinus* samples, the specificity, or the percentage of mapped reads that map to target regions, ranged from 24.4% to 27.7% with an average of 25.6%. Similar specificity was observed in the other chipmunk species: 27.6% in *T. amoenus*, 27.2% in *T. ruficaudus,* and 29.1% in *T. striatus* (Figure 2). 

Specificity levels are lower in our study compared to most studies in humans (50-75%, [7,21]). This decline likely has two principle causes. First, amplified, multiplexed libraries tend to show much lower specificity [22]. We see the same range of specificity across differing levels of multiplexing (Figure 2), indicating that this is not an issue of multiplexing *per se*, but rather specific to enriching amplified libraries with long complementary adapters. The use of a different barcoding system that enables pooling and hybridization prior to amplification would likely help reduce these effects. A second and likely larger issue is the fundamental limitation of not having an available reference genome. We calculated specificity following previous studies as the proportion of reads that map to the genome that fell within target regions. One important caveat in our data is that we observe a much larger proportion of un-mapped reads. We suspect that the observed low specificity is a reflection of reduced mapping efficiency in the absence of a complete reference genome. For example, among the reads mapped to assemblies corresponding to *T. alpinus* target exons, only ~4% of them were exclusively within flanking regions, compared to 32.6% in a recent human study [4]. Although high specificity is often obtained in single sample capture experiments, this approach is simply not cost-effective for population-level sampling [22] and the low overall specificity is more than compensated for by the ability to capture many individuals in parallel.

**Figure 3:**
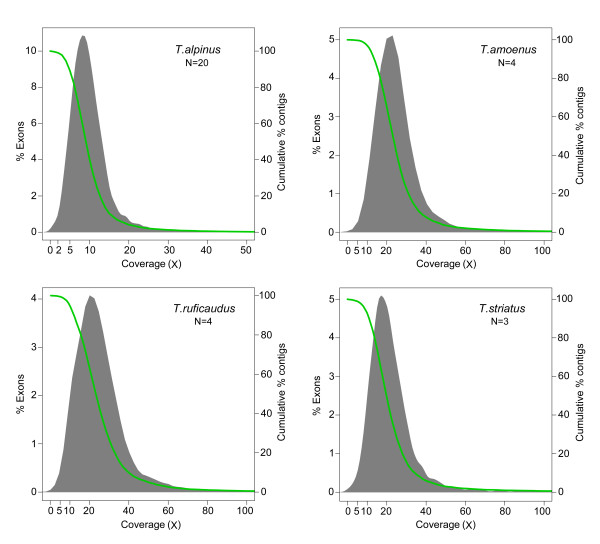
**Sequence coverage of target exons enriched in the four chipmunk species.** The columns show the distribution of average base coverage per exon. Coverage is shown on the X-axis, binned percentage of exons at each coverage on the Y-axis (left). The green line and right Y-axis show the cumulative coverage as a percent of total exons.

The average base coverage within target exons for individual *T. alpinus* libraries ranged from 7.1 to 13.7X, with a mean of 10.1X ( Additional file 5). 99.6% of the targets had a mean coverage of at least 1X, 89.3% had a mean coverage greater than 5X, and 40.3% had a mean coverage greater than 10X (Figure 3). The average coverage per lane for the other three *Tamias* species libraries was approximately two times greater than for *T. alpinus* (25.7X in *T. amoenus*, 25.9X in *T. ruficaudus,* and 22.7X in *T. striatus*), as expected because nearly twice as much sequence data were generated.

As expected, average coverage for the mitochondrial genome was at least one order of magnitude greater than for nuclear exons due to the higher per cell copy number of mtDNA. We also observed greater variance associated with sequence coverage among samples for the mitochondrial genome, which could reflect a difference in the amount of mitochondrial template present in each DNA sample ( Additional file 3: Figure S4; Additional file 5). Nevertheless, the distribution of coverage for aligned reads (valleys and peaks) along the entire mitochondrial genome showed great concordance across all four species, indicating that the performance of each capture experiment is consistent.

The comparison of sequence coverage among intended targets within each library was influenced by the base composition of the targets. We found that targets with exceptionally high or low G/C ratios led to low coverage ( Additional file 3: Figure S5), which may be due to poor annealing and secondary structure formation during the hybridization [21]. We found a strong correlation in terms of capture efficiency between the same targets among species libraries ( Additional file 3: Figure S6), as well as among samples within each species (data not shown), negating the existence of a species-specific capture bias. These results suggest that the performance of each target in different capture experiments is highly reproducible.

**Figure 4:**
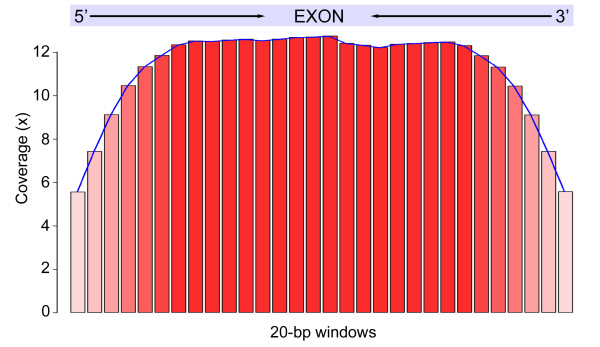
**Coverage-exon distance distributions.** Exons that ranged between 201–600 bp were used for generating the plot. Each target exon was split into 20-bp bins depicted by the red bar (X-axis). The average base coverage within each exon bin is shown on the Y-axis.

For each target exon, we found that the coverage among bases was mostly uniform except for at the edges. For example, base coverage for *T. alpinus* exons increased asymptotically from 5.5X at the 5’ and 3’ ends of exons towards the center were it reached ~12X and plateaued at 80–100 bp from the ends (Figure 4). A major limitation of transcriptome-based array design is the absence of tiling probes that span exon-intron boundaries, and fewer probes tiled at the ends of exons results in reduced coverage of contig edges. To accommodate this shortfall, we applied extra tiling probes to the edges of each exon to promote more uniform coverage. However, our results indicate that this method did not completely solve the expected “edge effect”. To address the edge effect completely, future array designs could use denser tiling even further from the edge and/or print duplicate probes to target the first or last few bases of each exon.

### Estimate of empirical error rates

Two important sources of errors in NGS analyses are sequencing (or base calling) error and contamination. Sequencing error, particularly on the Illumina platform, can be as high as ~1% [23]. Multiplexing samples can lead to cross sequence contamination caused by DNA contamination among specimens during lab work, swapping sample-specific barcodes during library preparation or bulk amplification of libraries (i.e., recombinant PCR), and/or mis-assigning the reads during the library de-multiplexing. Potential errors can also be derived from *de novo* assembly and alignment. These sources of errors can introduce bias into the variant calling based on exon capture data and result in false SNPs and hence, unreliable genotypes.

To address these related issues of exon capture and NGS sequencing, we targeted the complete mitochondrial genome, putative X-linked genes, and the Y-linked SRY gene on our capture arrays, while using both male and female specimens in the pooled genomic libraries. For males, these loci are haploid, and therefore, all variation within individuals should stem from sequencing or assembly errors. The *Spermophilus* SRY gene was captured in all male *Tamias* samples and was absent in all females except for one *T. striatus* female that had 6 reads that mapped to SRY. We identified a set of 26 putatively X-linked genes in *T. alpinus* for which we calculated overall error rate (the number of mismatched bases over the total number of aligned bases) in males to be 0.037%. The error rate for the *T. alpinus* mitochondrial genome was 0.045%, which is consistent with the X-linked loci. The results show that the empirical error rate for cleaned reads was comparable to that observed in DNA libraries of other species sequenced using the same platform (Singhal et al. unpublished), and corresponds to a Phred quality score between 33.5-34.3. We also calculated the average un-calibrated quality score of cleaned reads to be 36.3, indicating an error rate of 0.023%. This result suggests that the un-calibrated quality scores may under-estimate the true error rates of the sequence reads, which supports the notion that error rates estimated solely based on raw quality scores produced by NGS base-calling algorithms can be inaccurate [24]. The empirical error rate can be used to recalibrate raw quality scores and improve the accuracy of genotype calling [23]. Since genotype calling is not a focus of the present paper, we did not perform quality score re-calibration but related information can be found in [4] and [23].

### Divergence vs. capture efficiency

One hundred and sixty-two 1 kb genomic intervals of *I. tridecemlineatus* were targeted on the arrays. The sensitivity of this set of divergent targets dropped to ~90% for all *Tamias* species libraries, compared with a sensitivity of 99% when using *T. alpinus* exons as probes for target enrichment. Owing to the fact that we had 20 *T. alpinus* libraries sequenced on the same Illumina lane while the other 11 *Tamias* libraries were sequenced on two other lanes, the total data yield of each *T. alpinus* library was only about 1/4 that of other *Tamias* libraries. To eliminate this bias we randomly sub-sampled 5 million reads from each individual library and aligned them to the assemblies associated with targeted *T. alpinus* exons and the *I. tridecemlineatus* genomic intervals, respectively. The normalized average coverage for *T. alpinus* libraries was 10X. The average sequence divergence between the targeted *T. alpinus* exons and the other two western chipmunks, *T. amoenus* and *T. ruficaudus,* was 0.58%. The normalized coverage for *T. amoenus* and *T. ruficaudus* (12X) was slightly higher but still consistent with that of *T. alpinus.* The eastern chipmunk, *T. striatus*, has a slightly more divergent genome (1.5%) from *T. alpinus* yet the coverage for the two species was nearly identical ( Additional file 3: Figure S7). Vallender [25] applied human exome capture to non-human primates and suggested that the divergence cutoff for unbiased capture is around 4%. Our study clearly demonstrates that there is no decrease in capture efficiency within 1.5% divergence (such is the level of *T. striatus* to *T. alpinus*). According to these results, it is reasonable to postulate that unbiased sequence coverage could be obtained for all 23 chipmunk species across the western *Tamias* clade using the array designed from a single species transcriptome.

**Figure 5:**
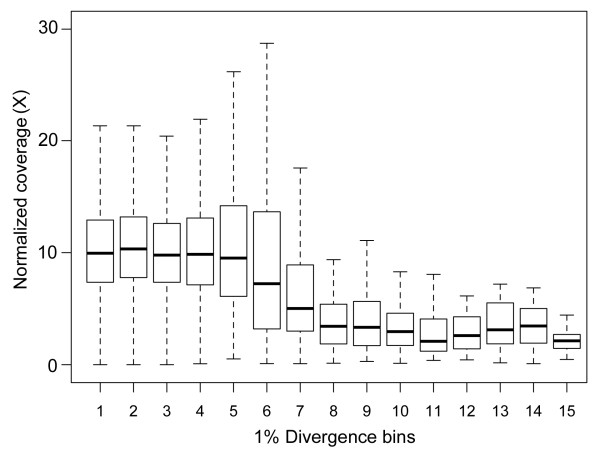
**Capture efficiency vs. sequence divergence.** The captured reads from all species libraries (*Tamias alpinus*, *T. amoenus*, *T. ruficaudus*, and *T. striatus*) derived from *T. alpinus* exon and *Ictidomys tridecemlineatus* genomic interval targets were combined to generate the plot. Outliers are not shown in the plot. Capture efficiency is represented by normalized base coverage. Sequence divergence between the targets and the corresponding in-target assemblies (X-axis) were placed in 1% bins.

As expected, there is an abrupt reduction in capture efficiency when using a divergent genome for capture array design. The sequence divergence between targeted *I. tridecemlineatus* genomic (presumably largely non-coding) intervals and genomes of the four *Tamias* species ranged from 8.76 to 8.98%. The results showed a 3 to 4-fold decrease in average sequence coverage among these regions ( Additional file 3: Figure S7), which support the finding by Vallender [25] that the level of coverage starts to decrease rapidly when the divergence becomes greater than 5% or more (Figure 5). Note that at least some of the un-captured *I. tridecemlineatus* regions are likely to be completely absent from the *Tamias* genome. Moreover, the sequence alignments between *Tamias* assemblies and the corresponding *I. tridecemlineatus* genomic intervals are dominated by extensive indels that would reduce the mapping efficiency around such regions, which could amplify the effect of local nucleotide difference on the level of coverage. Nevertheless, 90% of the *I. tridecemlineatus* intervals were still covered by reads at a mean coverage of 3-4X. The divergence between coding regions in *Tamias* and in *U. beldingi*, a close relative to *I. tridecemlineatus*, is around 5%. Non-coding regions are expected to be less conserved than protein-coding regions on average. Therefore, if only orthologous exons of *I. tridecemlineatus* were targeted we would reasonably expect an elevated capture efficiency with sequence coverage falling into the range of 4 to 12X along with higher sensitivity (>90%). 

### Orthologous markers and detection of candidate SNPs

The number of orthologous loci shared between *T. alpinus* and each of the other three chipmunk species are very similar, representing an approximately 3.6 Mb genotypic alignment. Overall, 10,544 orthologous loci with an average size of 339 bp were mutually shared among the four species. By aligning the reads of each species to the *T. alpinus* in-target assemblies, we found numerous SNPs between and within species, across all locations (intronic, UTR, and coding regions), and across both functional categories (non-synonymous, synonymous) (Table 1). We refrain from making too many conclusions based on these SNP calls here, as SNP calling is highly dependent on the read alignment software used and the variant calling algorithm. That said, our method demonstrates that transcriptome-based exon capture can facilitate the identification of a large number of orthologous markers with thousands of fixed differences among species of varying divergence. Pooling population samples on a single capture also provides a cost-effective method for SNP discovery that is invaluable for population genomic inferences of demography and selection.

**Table 1:** Summary of identified SNPs in orthologous loci

**SNP summary**^**a**^		***T. alpinus***	***T. amoenus***	***T. ruficaudus***	***T. striatus***
Fixed	Synonymous	-	5812	5579	13984
	Non-synonymous	-	1617	1616	3217
	Intron	-	2286	2222	2097
	UTR	-	523	515	447
	Total	-	10238	9932	19745
Polymorphic	Synonymous	880	2426	2625	1969
	Non-synonymous	512	860	892	633
	Intron	636	1052	1202	339
	UTR	173	277	284	77
	Total	2201	4615	5003	3018
Total difference	Synonymous (%)	880 (40.0)	8238 (55.5)	8204 (54.9)	15953 (70.1)
	Non-synonymous (%)	512 (23.2)	2477 (16.7)	2508 (16.8)	3850 (16.9)
	Intron (%)	636 (28.9)	3338 (22.5)	3424 (22.9)	2436 (10.7)
	UTR (%)	173 (7.9)	800 (5.4)	799 (5.3)	524 (2.3)
	Total (%)	2201 (100)	14853 (100)	14935 (100)	22763 (100)

^a^ Sequence reads of each species were aligned to the in-target assemblies of *T. alpinus* to estimate the level of genetic variability in the orthologous markers.

## Conclusions

Our goal for this study was to develop genomic resources for the application of NGS to a lineage lacking reference genomes, develop targets for exon capture, and test the performance of these new resources across phylogenetically divergent species. In summary, our strategy as it pertains to our study species, involved generating a *de novo* transcriptome for the chipmunk (*T. alpinus*), designing an array based on identified exons, pooling individually barcoded genomic libraries of a few moderately divergent species (*T. alpinus*, *T. amoenus*, *T. ruficaudus* and *T. striatus*) before exon capture, sequencing using multiplexed NGS, and applying bioinformatics approaches to analyze exon capture data without a reference genome.

The strategy developed here proved successful and will benefit future studies on multiple fronts: 1) Our capture array design is only based on transcriptome sequences from one individual specimen. Compared to whole genome sequencing, this is a much faster and economically viable approach for generating tens of thousands of genetic markers; 2) Multiplexing prior to capture is key to cost-effective population-level sequencing. The level of specificity, sensitivity, and read coverage of target regions is highly consistent among samples, indicating that pooling indexed libraries before exon-capture is a highly effective method; 3) The performance of each target is highly reproducible among independent capture experiments and the specificity is independent of the number of libraries that can be multiplexed on an array; 4) Base coverage is fairly even within exons, except at the edges. This edge effect could be further mitigated by extending the range of high density tiling baits at the ends of exonic regions, or by subsequent extension of probes into intronic regions of the assembled contigs recovered by initial captures; 5) Without having a prior reference genome, *de novo* assemblies of the captured reads can effectively provide a reference for mapping sequence reads, which allows for variant calling using existing tools; 6) Coverage does not decrease for species within 1.5% divergence in coding regions, but significantly declines when using more divergent targets (>8.7%) for array design. However, even then, a sensitivity of ~90% can be expected based on our results; 7) Over ten thousand orthologous loci and thousands of high quality candidate SNPs were identified that can be used for future population genomic and phylogenetic inference.

There are now diverse DNA capture methods available for non-model organisms, including PCR-generated bait capture to enrich specific loci from tens of samples [26,27] and DNA-hybridization based capture to target hundreds of loci from hundreds of samples. We suggest that studies should select the methods that best suit the questions at hand. For example, approaches that use ultraconserved element anchors (UCEs) [9,28] and anchored enrichment [6] have demonstrated great utility for tackling deeper phylogenetic nodes. Our study showcases the potential of a *de novo* transcriptome-enabled, multiplexed, exon capture method for sequencing thousands of orthologous loci over a vast number of non-model samples. Our approach can target exons spanning a wide range of evolutionary rates, thus it can be applied to population genomics for detecting selection and demography, species delimitation, and resolving phylogenies at low-moderate phylogenetic distance. Overall, adopting this cost-effective approach and associated analytical methods by ecological and evolutionary labs will expand the realm of possibility for addressing various evolutionary questions, ranging from populations to large assemblages of related species.

## Methods

### Samples

Three species of chipmunks from the western *Tamias* clade, *T. alpinus* (n=20), *T. amoenus* (n=4) and *T. ruficaudus* (n=4), and an eastern chipmunk, *T. striatus* (n=3), were collected for sequencing (Figure 1; Additional file 8). Tissues for DNA extraction were preserved in 90% ethanol or frozen. The specimen used for transcriptome sequencing was a male *T. alpinus* (MVZ224483) collected from Bullfrog Lake, Kings Canyon National Park, California in November 2009. Liver, kidney, spleen, and heart tissues were preserved in RNA*later* immediately following euthanasia and then archived at −80°C.

### Transcriptome sequencing

We used multiple tissues for RNA preparation in order to retrieve as many expressed transcripts as possible. Total RNA was extracted from each tissue using Qiagen RNeasy kits. The quantity and quality of total RNA was assayed using an Agilent Bioanalyzer 2100 and Nanodrop. Only RNA with a total amount ranging from 5–10 μg and an RNA integrity number (RIN) greater than 8 was used. Poly (A+) RNA was isolated from the total RNA by two cycles of purification using Sera-Mag® Magnetic Oligo(dT) magnetic beads. Resulting mRNA was quality verified as above and combined in equal molar ratios across tissues. Synthesis of cDNA and library preparation was performed as outlined in the Illumina mRNA sequencing sample preparation guide (rev. D) with slight modifications: we used 70°C instead of the recommended 94°C in order to avoid over-fragmentation of the mRNA and we size-selected the final library from 350–400 bp using an agarose gel extraction. We then sequenced the pooled library using one lane of 100 bp Illumina (GAIIx) paired-end sequencing at the QB3 research facilities at the University of California, Berkeley.

### Data filtration

In order to clean the raw sequence reads, we first removed identical forward and reverse reads. Duplicated reads are removed to avoid inflating coverage estimates and to decrease the computational burden for *de novo* assembly. We further trimmed unique reads by removing adapters, low complexity, and low quality (Q-score < 20) sequences using Blat [29], Trimmomatic (http://www.usadellab.org/cms/index.php?page=trimmomatic) and custom Perl scripts. We identified and removed any reads derived from contaminants by screening against the human and *Escherichia coli* genomes using Bowtie [30].

### *De novo* assembly, annotation, and exon identification

Cleaned reads were *de novo* assembled using ABySS [31] on the Texas Advanced Computing Center Ranger cluster (http://www.tacc.utexas.edu/). We generated individual assemblies under a wide range of k-mers (21, 31, 41, 51, and 61) and coverage values (2, 3, 5, and 10), obtained 20 raw assemblies, and then used cd-hit-est (http://weizhong-lab.ucsd.edu/cd-hit/), Blat, and CAP3 (http://seq.cs.iastate.edu/) to cluster and merge all raw assemblies into final, less-redundant assemblies.

The resulting *T. alpinus* transcriptome was then annotated using the BLASTX algorithm [32] and a database of human, mouse, rat, and thirteen-lined ground squirrel proteins with a minimum E-value of 1e-10. The exon-intron structure of each annotated *T. alpinus* transcript was identified using a 3-step strategy: *i*) BLASTX was used to search for correspondence between *T. alpinus* annotated transcripts and mouse proteins; *ii*) matched mouse proteins from step *i* were compared to mouse genome nucleotide sequence using exonerate (http://www.genome.iastate.edu/bioinfo/resources/manuals/exonerate/exonerate.man.html) -protein2genome in order to locate the exon-intron boundaries of each protein, and to determine the nucleotide sequence of each exon; *iii*) Exonerate -est2genome was then used to compare the sequences of mouse exons to each *T. alpinus* transcript to identify exon boundary coordinates.

### Array design

We used the Agilent SureSelect custom 1M-feature microarrays to target 11,975 nuclear exons (200 bp or greater) identified from the annotated *T. alpinus* transcriptome. These exons were derived from 6,249 annotated proteins with an overall target size of around 4 Mb. Probes were designed using customized scripts originally developed by Hernán Burbano and following the recommendations of Hodges et al. ([7]; see also [22]). Briefly, 60 bp probes were tiled at 4 bp intervals across individual exon targets. Typically in exome capture, probes are designed to extend beyond exon-intron boundaries to promote uniform read depth at both ends of the targets [7]. However, because we did not have genomic sequence for the introns, we used a 1 bp tiling strategy near exon ends to mitigate the effect of reduced coverage at the exon edge.

In addition to targeting the *T. alpinus* transcriptome, we targeted probes from four other sources. First, we used 2 bp tiling probes to capture 162 1 kb anonymous genomic intervals from the thirteen-lined ground squirrel (*I. tridecemlineatus*) draft genome (2X, Sanger sequencing). These regions were selected to test the robustness of probes designed from a divergent reference. Second, we used 764 20 bp tiling probes spanning non-repetitive regions of the ~16 kb *Tamias* complete mitochondrial genome, which enabled us to determine sequence contamination and empirical sequencing error rate. Third, we used 1 bp tiling probes targeting 350 bp of the consensus *Spermophilus* (*S. fulvusS. majorS. pygmaeus*) SRY gene. This Y-linked locus can be used as an additional control for cross-sample contamination in females. Finally, we targeted seven nuclear genes previously sequenced in *Tamias* (6,031 bp total), with 4 bp tiling: acrosin (1,558 bp), acp5 (361 bp), cmyc (896 bp), rag-1 (764 bp), anon (720 bp), zan (853 bp), and zp2 (879 bp). These genes represent the entirety of genomic data available in this group prior to our project [13,14] and were used as positive controls in a post-capture qPCR assay to determine the initial enrichment quality.

In order to avoid nonspecific hybridization of genomic DNA to the capture arrays, we employed a soft-masking approach [7] to exclude probes that likely contain highly repetitive elements. Specifically, we combined the *T. alpinus* transcriptome and *the I. tridecemlineatus* genome to create a single genome set as a reference. We then calculated the frequency of all 15-mers on both strands of the reference and excluded probes containing 15-mers found at a frequency of 50 or greater. This cut-off is arbitrary and intentionally more stringent than commonly used given the inherent limitations expected when relying upon a divergent and poor quality reference as the basis for soft-masking.

### Genomic DNA library preparation and multiplexing

Genomic DNA was extracted from liver or muscle tissues of *T. alpinusT. amoenusT. ruficaudus,* and *T. striatus* specimens using Qiagen DNeasy Blood & Tissue kits. DNA was sheared using a Bioruptor with 4–6 rounds of sonication (7 minutes per round on high, 30s on 30s off). The ideal DNA fragment size distribution should range from approximately 100–500 bp, with a mean of 200–250 bp. Individual genomic libraries were prepared following the protocol outlined by [33] with slight modifications. This protocol describes a fast, simple, and cost-effective method for preparing indexed genomic libraries for Illumina. Briefly, universal adapters are ligated to each library, and then the libraries are indexed via PCR using 7-nt barcoded primers prior to exon capture. Each PCR indexed library was pooled by species in equimolar ratios to obtain 20 μg total DNA for each hybridization experiment.

### Exon capture

We used one microarray for each pooled set of species-specific libraries. For example, 20 pooled *T. alpinus* libraries were hybridized on one array. We followed the detailed procedure for array-based exon capture that is described in [7] with slight modifications by [33]. Each genomic library was denatured in a solution containing excess blocking oligos and Cot-1 DNA. *Tamias* Cot-1 DNA was isolated following [34].

The pooled libraries were then hybridized in a SciGene 777 microarray hybridization oven at 65°C for 65 hours, rinsed, and eluted at 95°C. The eluted DNA was then PCR amplified for 14 cycles, reflecting the number of cycles expected to produce the optimal yield as empirically estimated from the starting template concentration. This precaution was taken to ensure that we did not over-amplify libraries and increase the likelihood of barcode swapping between fragments and the formation of hetero-duplexes [35]. We verified target enrichment with qPCR analysis of amplified elute, following [7]. Three target and non-target regions were evaluated independently.

We then diluted each library to a final concentration of 10 nM in 10 μl. Sequencing was performed using the Illumina HiSeq 2000 platform (100 bp paired end) provided by the QB3 at UC Berkeley. The library consisting of 20 *T. alpinus* was sequenced on one lane, while the 4 *T. amoenus*, 4 *T. ruficaudus,* and 3 *T. striatus* libraries were equally combined and sequenced on two lanes.

### Genomic sequence data cleanup and *de novo* assemblies

Sequences were assigned to individuals based on their unique barcodes and filtered as described above. ABySS and SOAPdenovo [36] were used to assemble cleaned reads of each of the four species libraries. For example, 20 *T. alpinus* libraries were assembled together to make species-specific consensus assemblies. Assembly using multiple kmer sizes was performed. Twenty raw assemblies were obtained with ABySS by setting the k-mers to 21, 31, 41, 51, and 61, and k-cov to 6, 10, 15, and 20. SOAPdenovo did not allow adjustment of k-cov so we only set the k-mers to 21, 31, 41, 51, and 61 (5 raw assemblies). Twenty-five raw assemblies from each species library were further merged using the same methods described above. This produced one consensus assembly for each of the four species.

### Evaluation of capture efficiency

The following parameters were used to evaluate the performance of the exon capture results: i) sensitivity, or the percentage of the intended targets covered by sequence reads; ii) specificity, or the percentage of sequence reads aligned to selected targets; iii) sequence or base coverage, or the number of reads aligned to a base within a target sequence or a particular base; iv) uniformity, or the variance of the average read depth among different targets and within targets; and v) reproducibility, or the consistency of results obtained from replicated capture experiments.

We used reciprocal BLAST to associate contigs in the consensus assemblies with the target exons and estimate the exon capture sensitivity. To improve mapping efficiency, we did not use the *T. alpinus* transcriptome as a reference for alignment. Instead, we used the contigs in the consensus assemblies that were best matches to targets, or in-target assemblies, as our reference. These contigs were annotated for exon-intron structure. The sequence reads of each individual library were then mapped to its own species consensus assemblies as well as in-target assemblies using Novoalign (http://www.novocraft.com). The exon capture specificity was calculated by dividing the number of reads aligned to targets by the total number of reads aligned to the consensus assemblies. Only reads that mapped to unique locations in the in-target assemblies were used to calculate specificity. Novoalign output was parsed using SAMtools [37], and the ‘mpileup’ function in SAMtools was used to estimate coverage. Uniformity was measured by comparing average sequence coverage among targets, as well as the average base coverage within each target. The level of uniformity could also be estimated by comparing enrichment efficiency for the same targets across different species libraries. Since each species library was captured on independent arrays, the level of uniformity is reflective of the reproducibility. If the sequence coverage of a given target is correlated among libraries and capture arrays then we believe that the enrichment is uniform and reproducible across regions.

### Estimating empirical error rates

We compared each of the captured *Tamias* mitochondrial consensus genomes to the mouse complete mitochondrial genome obtained from NCBI (JQ003190.1), and arranged the sequences in the order that genes/regions occur in the mitochondrial genome. We aligned reads to the captured mitochondrial consensus, parsed the alignments and identified the mismatches. The error rates were calculated by dividing the observed total number of mismatches by the total number of aligned bases. Likewise, we also determined the empirical level of errors for putatively X-linked genes in the libraries of *Tamias* males, for which we should observe no polymorphism.

Putatively X-linked genes were identified by: i) using the Ensembl BioMart tools to search non-redundant, conserved orthologs on X chromosomes of human, dog (*Canis familiaris*), rat and mouse; ii) these conserved orthologs were searched against *T.alpinus* exons with BLAST; iii) matched exons with female to male average sequence coverage ratios ranging from 1.9-2.1 were selected as putatively X-linked. The level of errors was also assessed based on the proportion of reads mapped to the Y-linked SRY gene in females.

### Capture efficiency estimate of array designed from a divergent genome

We anonymously selected 162 genomic intervals of the thirteen-lined ground squirrel (*I. tridecemlineatus*) to hybridize with genomes of the four *Tamias* species. The methods used for searching in-target assemblies, and measuring sensitivity and sequence coverage of the targeted regions were identical to the ones described above. Sequence divergence was calculated by dividing the number of observed mismatches by the number of alignable bases in the alignment between in-target contigs with the corresponding *I. tridecemlineatus* genomic intervals. The divergence between the targeted *T. alpinus* exons and in-target contigs of each species was measured using the alignment mismatches within exonic regions. We focused on the relationship between average sequence coverage and sequence divergence in different chipmunk species libraries when using *I. tridecemlineatus* (divergent) and *T. alpinus* (closely related) genomes to design arrays. All analyses and graphing was conducted in R [38].

### Identification of orthologous loci and preliminary SNP detection

We used reciprocal BLAST searches to compare in-target assemblies of each species to search for orthologous markers that were enriched among the four species. We aligned the reads of each species to the same reference (in-target assemblies of *T. alpinus*) to estimate the level of genetic variability of these markers. We did not align reads directly to the *T. alpinus* transcriptome in order to maximize mapping efficiency. Furthermore, mapping reads to species-specific *de novo* assemblies not only helps identify SNPs located within exons, but also identifies those located in flanking intronic and UTR regions. Candidate homozygous SNPs (fixed differences between species) and heterozygous SNPs (polymorphic within species) were determined from the reference sequences. Variant calling was conducted using SAMtools and SNPs were filtered via samtools.pl (varFilter -d 20 -D 5000). Reads that aligned to multiple reference regions were discarded, and sites containing mismatches were kept only if the coverage was 20X or greater. We also retained only high quality variable sites by filtering out sites with a Phred-scaled quality score of less than 30. The types of SNPs (synonymous, non-synonymous, intronic, 5’ and 3’ UTRs) were determined by comparison with the combined human, mouse, rat and thirteen-lined ground squirrel protein reading frames using bl2seq and BLASTX.

## Competing interests

The authors declare that they have no competing interest.

## Authors’ contributions

CM, JG and KB conceived the study. JG designed the capture experiment. KB and DV carried out laboratory experiments. KB and TL analyzed the data with help from other authors. SS and KB constructed the bioinformatics pipelines for RNA-seq and exon capture data. KB wrote the manuscript with comments from all other authors. All authors read and approved the final manuscript.

## Supplementary Material

Additional file 1 Cost-effectiveness of transcriptome-based exon capture for population genomic and phylogenetic applications. Click here for file

Additional file 2 Summary statistics for raw assemblies of transcriptome sequence data. Click here for file

Additional file 3 **Supplementary figures (Figures S1-S7).** Figure S1. Correlation between the length of annotated transcripts and the average base coverage. Figure S2. Divergence of orthologous transcripts between *Tamias alpinus* and *Urocitellus beldingi*. The sequence divergence between target exons selected in the present study and their orthologous genes in *Urocitellus beldingi* (red) follows a similar distribution pattern to that of all identified orthologous pairs between the two species (black). Figure S3. Raw assemblies of captured sequences from the four chipmunk species. The raw assemblies generated by ABySS and by SOAP*denovo* were combined for the summary. The mean and median lengths, and N50 of raw assemblies generated using various combinations of k-mer and k-cov were similar among species. Black bars indicate standard deviation. Figure S4. Sequence coverage of captured mitochondrial genome from each chipmunk species. The genes/regions of each *Tamias* mitochondrial contig were re-organized by comparing them to the reference mouse (*Mus musculus*) mitochondrial genome obtained from NCBI (JQ003190.1). The top horizontal bar depicts gene annotations except for the tRNA genes. A. *Tamias alpinus*; B. *T. amoenus*; C. *T. ruficaudus*; D. *T. striatus*. Figure S5. Impact of variable GC content of exons on the capture efficiency. Capture efficiency is shown by average base coverage within each target exon. Figure S6. Comparison of performance of exon capture in independent capture experiments. Exon base coverage was log transformed and is shown on the X- and Y-axis. There is strong correlation in capture efficiency between the same targets in different species libraries, captured on separate arrays. Figure S7. Sequence divergence (A) vs. normalized base coverage (B) in divergent and closely related targets. *TA*: *Tamias alpinus*; *TM*: *T. amoenus*; *TR*: *T. ruficaudus*; *TS*: *T. striatus*; *IT*: *Ictidomys tridecemlineatus*. TA: Target exons of *T. alpinus*; IT: Target genomic regions of *I. tridecemlineatus*. There is a significant drop in capture efficiency (normalized base coverage) of *I. tridecemlineatus* genomic targets compared to *Tamias alpinus* target exons. * *p*<0.001. Click here for file

Additional file 4 Statistics for de-multiplexing results of sequencing reads in each species library. Click here for file

Additional file 5 Exon capture data filtration and summary of mapping results. Click here for file

Additional file 6 Summary of raw assemblies for exon capture data. Click here for file

Additional file 7 Comparison of species-specific in-target assemblies and target exons. Click here for file

Additional file 8 Sample information. Click here for file

## References

[B1] Shendure J Hanlee J Next-generation DNA sequencing Nat Biotech 2008 26 1135 1145 10.1038/nbt1486 18846087

[B2] Good JM Reduced representation methods for subgenomic enrichment and next-generation sequencing Methods Mol Biol 2011 772 85 103 22065433 10.1007/978-1-61779-228-1_5

[B3] Gilad Y Pritchard JK Thornton K Characterizing natural variation using next- generation sequencing technologies Trends Genet 2009 25 463 471 10.1016/j.tig.2009.09.003 19801172 PMC3994700

[B4] Yi X Liang Y Huerta-Sanchez E Jin X Cuo ZX Pool JE Xu X Jiang H Vinckenbosch N Korneliussen TS Zheng H Liu T He W Li K Luo R Nie X Wu H Zhao M Cao H Zou J Shan Y Li S Yang Q Asan Ni P Tian G Xu J Liu X Jiang T Wu R Zhou G Tang M Qin J Wang T Feng S Li G Huasang Luosang J Wang W Chen F Wang Y Zheng X Li Z Bianba Z Yang G Wang X Tang S Gao G Chen Y Luo Z Gusang L Cao Z Zhang Q Ouyang W Ren X Liang H Zheng H Huang Y Li J Bolund L Kristiansen K Li Y Zhang Y Zhang X Li R Li S Yang H Nielsen R Wang J Wang J Sequencing of 50 human exomes reveals adaptation to high altitude Science 2010 329 75 78 10.1126/science.1190371 20595611 PMC3711608

[B5] Smith SA Wilson NG Goetz FE Feehery C Andrade SCS Rouse GW Giribet G Dunn CW Resolving the evolutionary relationships of molluscs with phylogenomic tools Nature 2011 480 364 367 10.1038/nature10526 22031330

[B6] Lemmon A Emme S Lemmon E Anchored hybrid enrichment for massively high-throughput phylogenomics Syst Biol 2012 10.1093/sysbio/sys049 22605266

[B7] Hodges E Rooks M Xuan Z Bhattacharjee A Benjamin Gordon D Brizuela L Richard McCombie W Hannon GJ Hybrid selection of discrete genomic intervals on custom-designed microarrays for massively parallel sequencing Nat Protoc 2009 4 960 974 10.1038/nprot.2009.68 19478811 PMC2990409

[B8] Hohenlohe PA Amish SJ Catchen JM Allendorf FW Luikart G Next-generation RAD sequencing identifies thousands of SNPs for assessing hybridization between rainbow and westslope cutthroat trout Mol Ecol Res 2011 11 117 122 10.1111/j.1755-0998.2010.02967.x 21429168

[B9] Faircloth BC McCormack JE Crawford NG Harvey MG Brumfield RT Glenn TC Ultraconserved elements anchor thousands of genetic markers spanning multiple evolutionary timescales Syst Biol 2012 10.1093/sysbio/sys004 22232343

[B10] Cosart T Beja-Pereira A Chen S Ng SB Shendure J Luikart G Exome-wide DNA capture and next generation sequencing in domestic and wild species BMC Genomics 2011 12 347 10.1186/1471-2164-12-347 21729323 PMC3146453

[B11] Gnirke A Melnikov A Maguire J Rogov P LeProust EM Brockman W Fennell T Giannoukos G Fisher S Russ C Gabriel S Jaffe DB Lander ES Nusbaum C Solution hybrid selection with ultra-long oligonucleotides for massively parallel targeted sequencing Nat Biotechnol 2009 27 182 189 10.1038/nbt.1523 19182786 PMC2663421

[B12] George RD McVicker G Diederich R Ng SB MacKenzie AP Swanson WJ Shendure J Thomas JH Trans genomic capture and sequencing of primate exomes reveals new targets of positive selection Genome Res 2011 21 1686 1694 10.1101/gr.121327.111 21795384 PMC3202285

[B13] Good JM Hird S Reid N Demboski JR Steppan SJ Martin-Nims TR Sullivan J Ancient hybridization and mitochondrial capture between two species of chipmunks Mol Ecol 2008 17 1313 1327 10.1111/j.1365-294X.2007.03640.x 18302691

[B14] Reid N Demboski JR Sullivan J Phylogeny estimation of the radiation western American chipmunk (Tamias) in the face of introgression using reproductive protein genes Syst Biol 2012 61 44 62 10.1093/sysbio/syr094 21878471 PMC3243737

[B15] Surget-Groba Y Montoya-Burgos JI Optimization of de novo transcriptome assembly from next-generation sequencing data Genome Res 2010 20 1432 1440 10.1101/gr.103846.109 20693479 PMC2945192

[B16] Robertson G Schein J Chiu R Corbett R Field M Jackman SD Mungall K Lee S Okada HM Qian JQ Griffith M Raymond A Thiessen N Cezard T Butterfield YS Newsome R Chan SK She R Varhol R Kamoh B Prabhu AL Tam A Zhao Y Moore RA Hirst M Marra MA Jones SJ Hoodless PA Birol I De novo assembly and analysis of RNA-seq data Nat Methods 2010 7 909 912 10.1038/nmeth.1517 20935650

[B17] Zhao QY Wang Y Kong YM Luo D Li X Hao P Optimizing de novo transcriptome assembly from short-read RNA-Seq data: a comparative study BMC Bioinformatics 2011 12 S2 10.1186/1471-2105-12-S14-S2 PMC3287467 22373417

[B18] Wall PK Leebens-Mack J Chanderbali AS Barakat A Wolcott E Liang H Landherr L Tomsho LP Hu Y Carlson JE Ma H Schuster SC Soltis DE Soltis PS Altman N dePamphilis CW Comparison of next generation sequencing technologies for transcriptome characterization BMC Genomics 2009 10 347 10.1186/1471-2164-10-347 19646272 PMC2907694

[B19] Bainbridge MN Wang M Burgess DL Kovar C Rodesch MJ D'Ascenzo M Kitzman J Wu YQ Newsham I Richmond TA Jeddeloh JA Muzny D Albert TJ Gibbs RA Whole exome capture in solution with 3 Gbp of data Genome Biol 2010 11 R62 10.1186/gb-2010-11-6-r62 20565776 PMC2911110

[B20] Li Y Vinckenbosch N Tian G Huerta-Sanchez E Jiang T Jiang H Albrechtsen A Andersen G Cao H Korneliussen T Grarup N Guo Y Hellman I Jin X Li Q Liu J Liu X Sparsø T Tang M Wu H Wu R Yu C Zheng H Astrup A Bolund L Holmkvist J Jørgensen T Kristiansen K Schmitz O Schwartz TW Zhang X Li R Yang H Wang J Hansen T Pedersen O Nielsen R Wang J Resequencing of 200 human exomes identifies an excess of low-frequency non-synonymous coding variants Nat Genet 2010 42 969 972 10.1038/ng.680 20890277

[B21] Mamanova L Coffey AJ Scott CE Kozarewa I Turner EH Kumar A Howard E Shendure J Turner DJ Target-enrichment strategies for next-generation sequencing Nat Methods 2010 7 111 118 10.1038/nmeth.1419 20111037

[B22] Burbano HA Hodges E Green RE Briggs AW Krause J Meyer M Good JM Maricic M Johnson PLF Xuan Z Rooks M Bhattacharjee A Brizuela L Albert FW de la Rasilla M Fortea J Rosas A Lachmann M Hannon GJ Pääbo S Targeted investigation of the Neandertal genome by array-based sequence capture Science 2010 328 723 725 10.1126/science.1188046 20448179 PMC3140021

[B23] Nielsen R Paul JS Albrechtsen A Song YS Genotype and SNP calling from next-generation sequencing data Nat Rev Genet 2011 12 443 451 10.1038/nrg2986 21587300 PMC3593722

[B24] Li R Li Y Fang X Yang H Wang J Kristiansen K Wang J SNP detection for massively parallel whole-genome resequencing Genome Res 2009 19 1124 1132 10.1101/gr.088013.108 19420381 PMC2694485

[B25] Vallender EJ Expanding whole exome resequencing into non-human primates Genome Biol 2011 12 R87 10.1186/gb-2011-12-9-r87 21917143 PMC3308050

[B26] Maricic T Whitten M Pääbo S Multiplexed DNA sequence capture of mitochondrial genomes using PCR products PLoS One 2010 5 e14004 10.1371/journal.pone.0014004 21103372 PMC2982832

[B27] Mason VC Li G Helgen KM Murphy WJ Efficient cross-species capture hybridization and next-generation sequencing of mitochondrial genomes from noninvasively sampled museum specimens Genome Res 2011 21 1695 1704 10.1101/gr.120196.111 21880778 PMC3202286

[B28] McCormack JE Faircloth BC Crawford NG Gowaty PA Brumfield RT Glenn TC Ultraconserved elements are novel phylogenomic markers that resolve placental mammal phylogeny when combined with species-tree analysis Genome Res 2012 22 746 754 10.1101/gr.125864.111 22207614 PMC3317156

[B29] Kent WJ BLAT-the BLAST-like alignment tool Genome Res 2002 12 656 664 11932250 10.1101/gr.229202 PMC187518

[B30] Langmead B Trapnell C Pop M Salzberg SL Ultrafast and memory-efficient alignment of short DNA sequences to the human genome Genome Biol 2009 10 R25 10.1186/gb-2009-10-3-r25 19261174 PMC2690996

[B31] Birol İ Jackman SD Nielsen CB Qian JQ Varhol R Stazyk G Morin RD Zhao Y Hirst M Schein JE Horsman DE Connors JM Gascoyne RD Marra MA Jones SJ De novo transcriptome assembly with ABySS Bioinformatics 2009 25 2872 2877 10.1093/bioinformatics/btp367 19528083

[B32] Altschul SF Madden TL Schaffer AA Zhang J Zhang Z Miller W Lipman DJ Gapped BLAST and PSI-BLAST: A new generation of protein database search programs Nucl Acids Res 1997 25 3389 3402 10.1093/nar/25.17.3389 9254694 PMC146917

[B33] Meyer M Kircher M Illumina sequencing library preparation for highly multiplexed target capture and sequencing Cold Spring Harb Protoc 2010 10.1101/pdb.prot5448 20516186

[B34] Trifonov VA Vorobieva NN Rens W Liehr T FISH With and Without COT1 DNA Fluorescence In Situ Hybridization (FISH): Application Guide 2009 Springer 99 109

[B35] Ruano G Kidd KK Modeling of heteroduplex formation during PCR from mixtures of DNA templates PCR Methods Appl 1992 2 112 116 1362127 10.1101/gr.2.2.112

[B36] Li R Zhu H Ruan J Qian W Fang X Shi Z Li Y Li S Shan G Kristiansen K Li S Yang H Wang J Wang J De novo assembly of human genomes with massively parallel short read sequencing Genome Res 2010 20 265 272 10.1101/gr.097261.109 20019144 PMC2813482

[B37] Li H Handsaker B Wysoker A Fennell T Ruan J Homer N Marth G Abecasis G Durbin R 1000 Genome Project Data Processing Subgroup: The sequence alignment/map (SAM) format and SAMtools Bioinformatics 2009 25 2078 2079 10.1093/bioinformatics/btp352 19505943 PMC2723002

[B38] R Development Core Team R: A language and environment for statistical computing. R foundation for statistical computing, Vienna, Austria 2011 http://www.R-project.org/

