# Supplementary material for: Transcriptome-based exon capture enables highly cost-effective comparative genomic data collection at moderate evolutionary scales
Source: BMC Genomics. 2012 Aug 17;13:403. doi: 10.1186/1471-2164-13-403 (PMC3472323; doi:10.1186/1471-2164-13-403)
Supplement: Additional file 1 — Cost-effectiveness of transcriptome-based exon capture for population genomic and phylogenetic applications. [file 1471-2164-13-403-S1.docx]

**Cost-effectiveness of transcriptome-based exon capture for population genetic and phylogenetic applications**

Our approach is suitable for population-level genomic scans in which both large sample size and genome-wide markers are preferable for inferring selection and demography. Each 1M SureSelect capture array costs only $700. Using the protocol outlined in [1] and [2], we estimated that the cost of reagents in library preparation and exon capture is ~$30 per individual. We demonstrated that capture performance is highly consistent among samples when multiplexing 20 individual libraries on a single array, however the number of multiplexed individual libraries on each array can be scaled up to 50 [3]. Including the cost of initial transcriptome sequencing, we estimate that the investment for a project involving 100 samples and 10,000 loci (total size of 4 Mb) is around $8,138 or $81 per individual. Using the current illumina HiSeq2000 sequencing platform, 30-35 Gb of raw sequence reads can be expected from one lane of 100 bp pair-end sequencing (as of April 2012, qb3). By taking the data loss during read filtration and mapping efficiency into consideration, the average per individual coverage is expected to be 16.5-20X. This level of sequencing effort is necessary for confidently filtering sequencing errors and other uncertainties related to genotype and SNP calling [4]. For a typical population genomic study, a genome scan using a large sample size sequenced at shallower coverage achieves higher statistical power than a small number of samples sequenced at deeper coverage [5]. Accordingly, if a project aimed for 5X coverage per individual for 400 samples at 10,000 loci (4 Mb), the cost would be ~$53 for each individual. Overall, beyond its unmatchable labor- and cost-effectiveness over traditional PCR and Sanger sequencing, the per individual cost using our approach is comparable to a project using solution-based hybrid enrichment involving the same sample size and fewer (up to 500) enriched nuclear markers[6].

Reference:

1. Meyer M, Kircher M: **Illumina sequencing library preparation for highly multiplexed target capture and sequencing.** *Cold Spring Harb Protoc* 2010, **2010**: doi:10.1101/pdb.prot5448.

2. Hodges E, Rooks M, Xuan Z, Bhattacharjee A, Benjamin Gordon D, Brizuela L, Richard McCombie W, Hannon GJ: **Hybrid selection of discrete genomic intervals on custom-designed microarrays for massively parallel sequencing.** *Nat Protoc* 2009, **4**: 960-974.

3. Burbano HA, Hodges E, Green RE, Briggs AW, Krause J, Meyer M, Good JM, Maricic M, Johnson PLF, Xuan Z, Rooks M, Bhattacharjee A, Brizuela L, Albert FW, de la Rasilla M, Fortea J, Rosas A, Lachmann M, Hannon GJ, Pääbo S: **Targeted investigation of the Neandertal genome by array-based sequence capture.** *Science* 2010, **328**: 723-725.

4. Nielsen R, Paul JS, Albrechtsen A, Song YS: **Genotype and SNP calling from next-generation sequencing data.** *Nat Rev Genet* 2011, **12**:443-451.

5. Kim SY, Li Y, Guo Y, Li R, Holmkvist J, Hansen T, Pedersen O, Wang J, Nielsen R: **Design of association studies with pooled or un-pooled next-generation sequencing data.** *Genet Epidemiol* 2010, 34:479-491.

6. Lemmon A, Emme S, Lemmon E: **Anchored hybrid enrichment for massively high-throughput phylogenomics**. *Syst Biol*, in press.
