# Supplementary figures and images for: Transcriptome-based exon capture enables highly cost-effective comparative genomic data collection at moderate evolutionary scales
Source: BMC Genomics. 2012 Aug 17;13:403. doi: 10.1186/1471-2164-13-403 (PMC3472323; doi:10.1186/1471-2164-13-403)

**
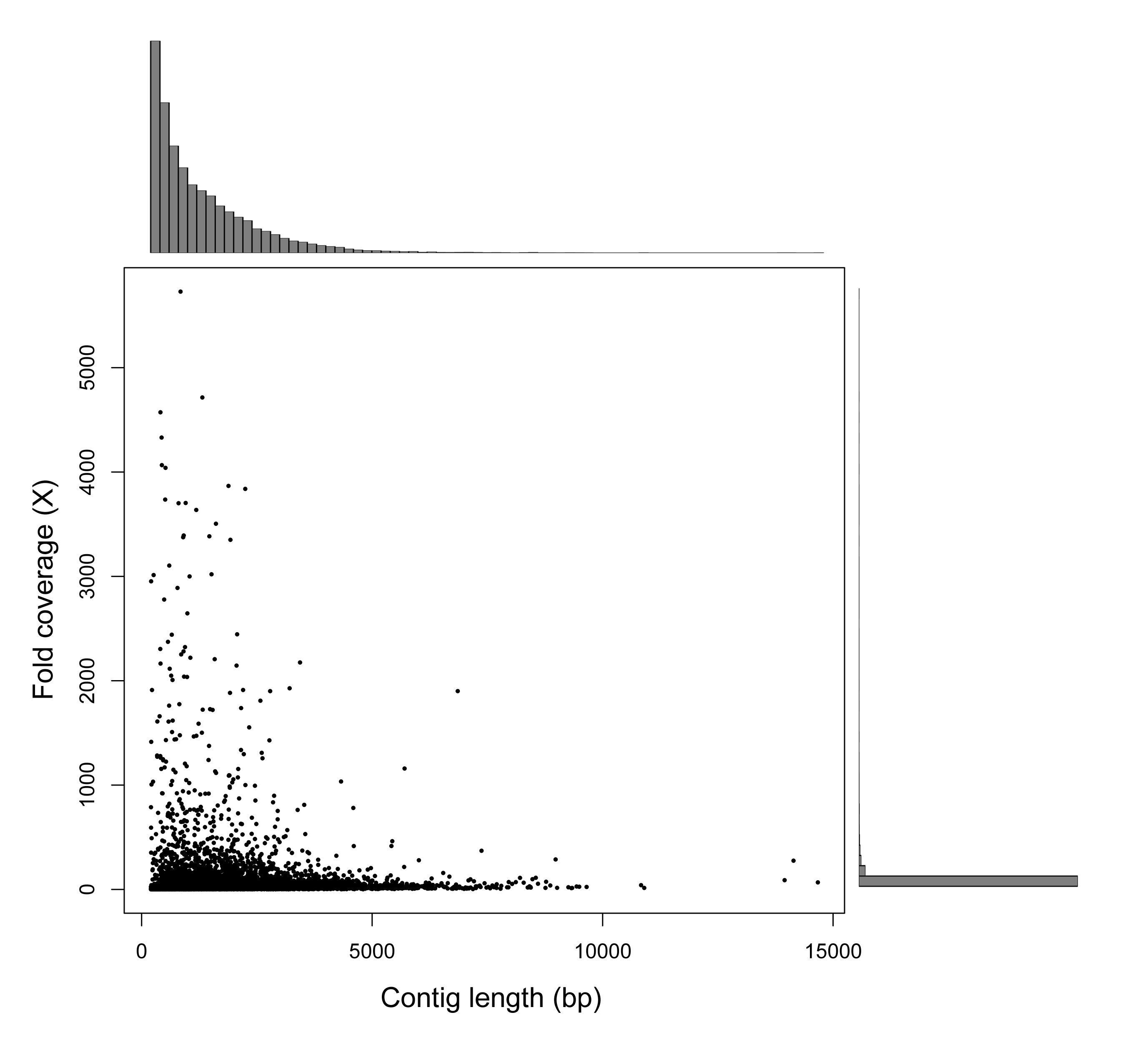
**

**Figure S1**

**
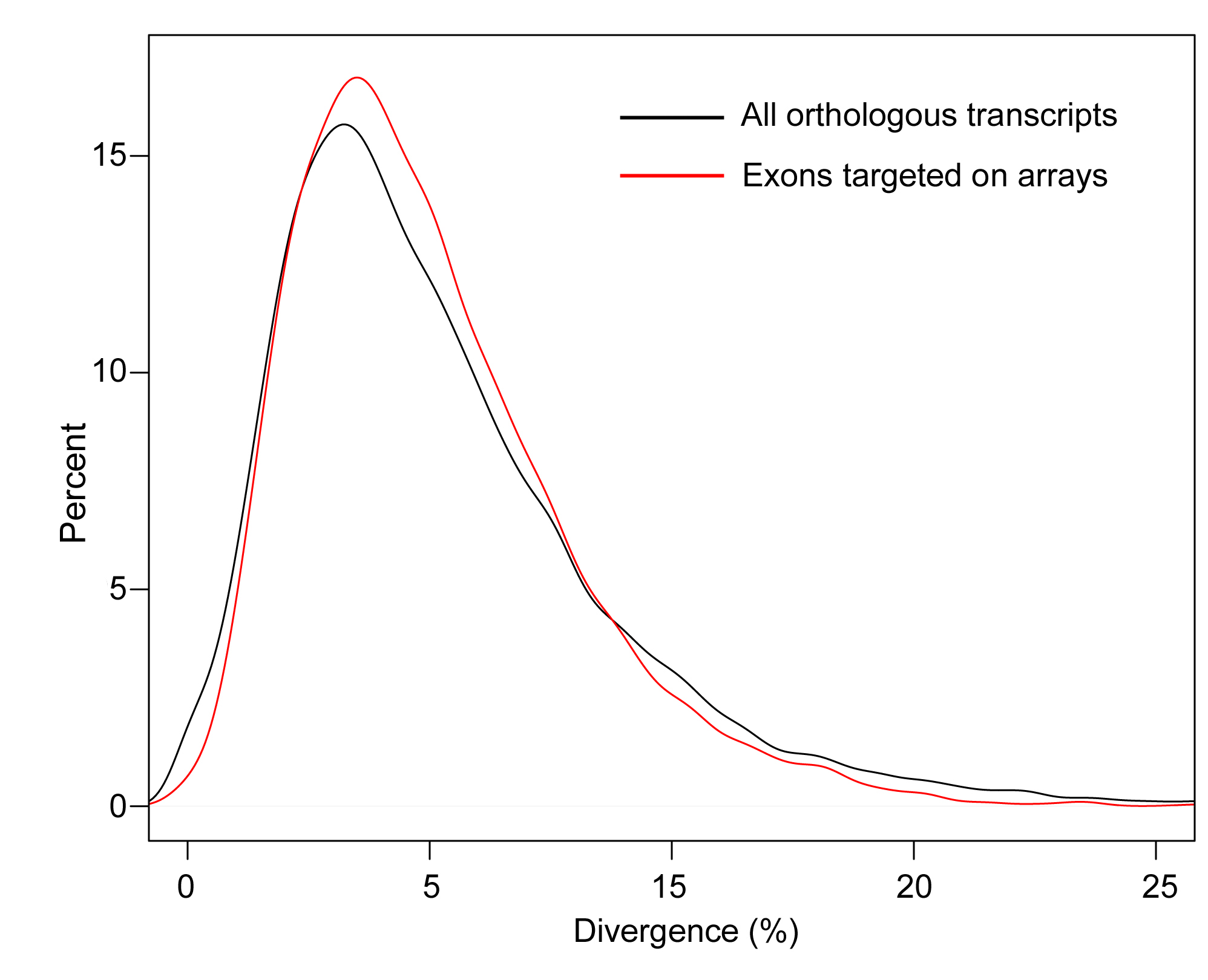
Figure S2**

**
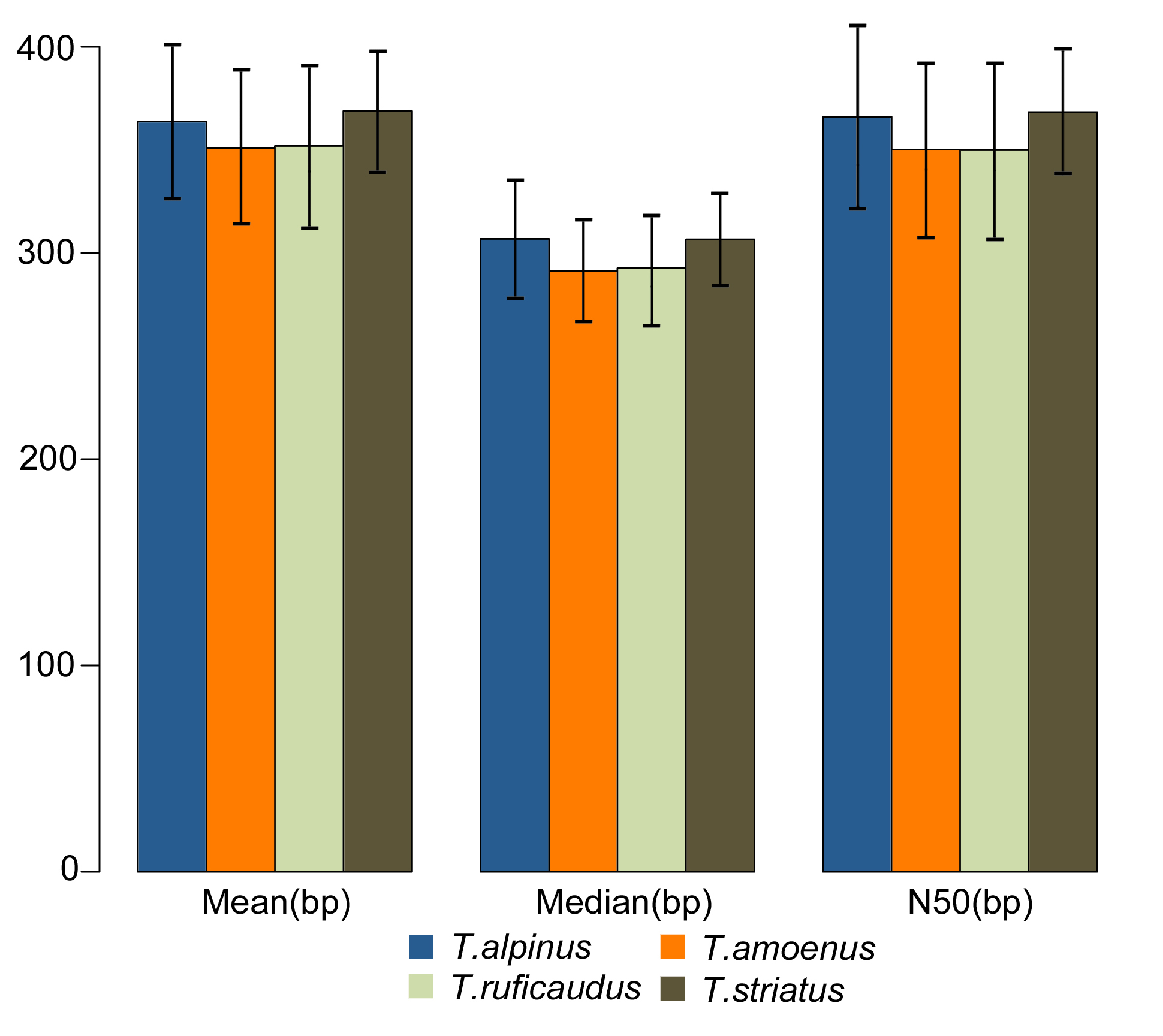
**

**Figure S3**

**
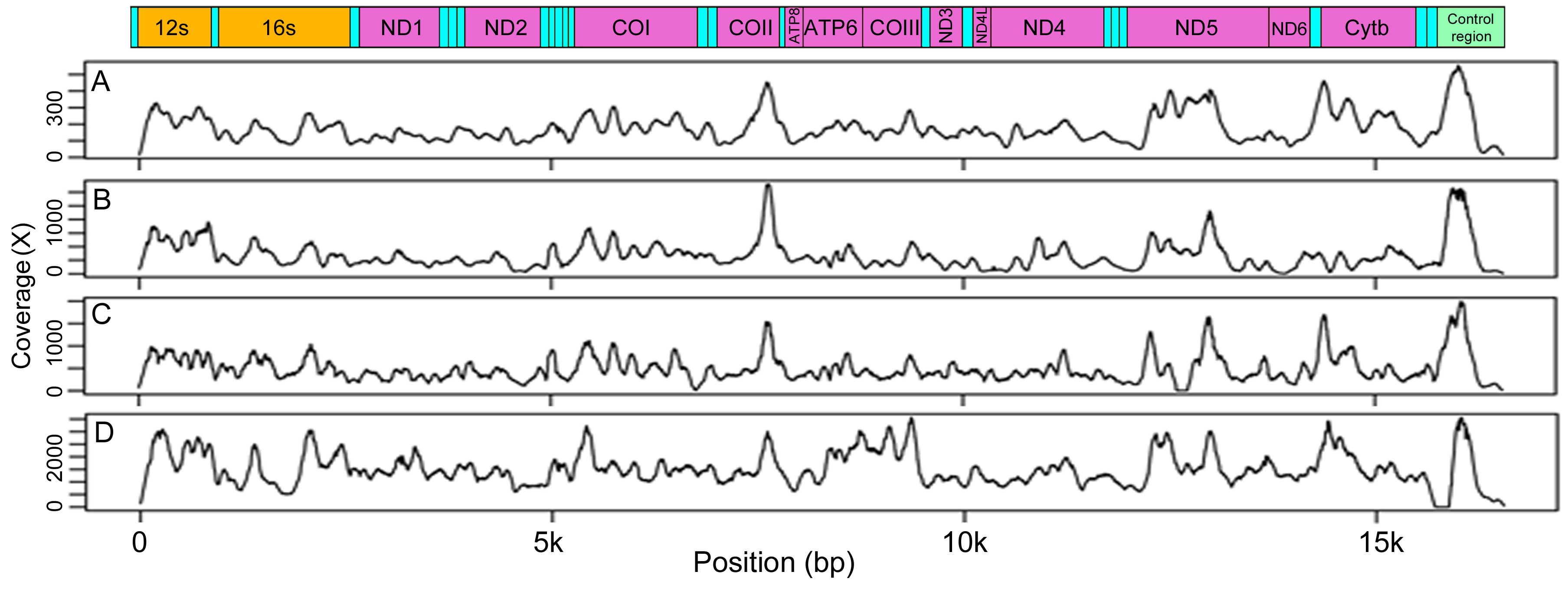
**

**Figure S4**

**
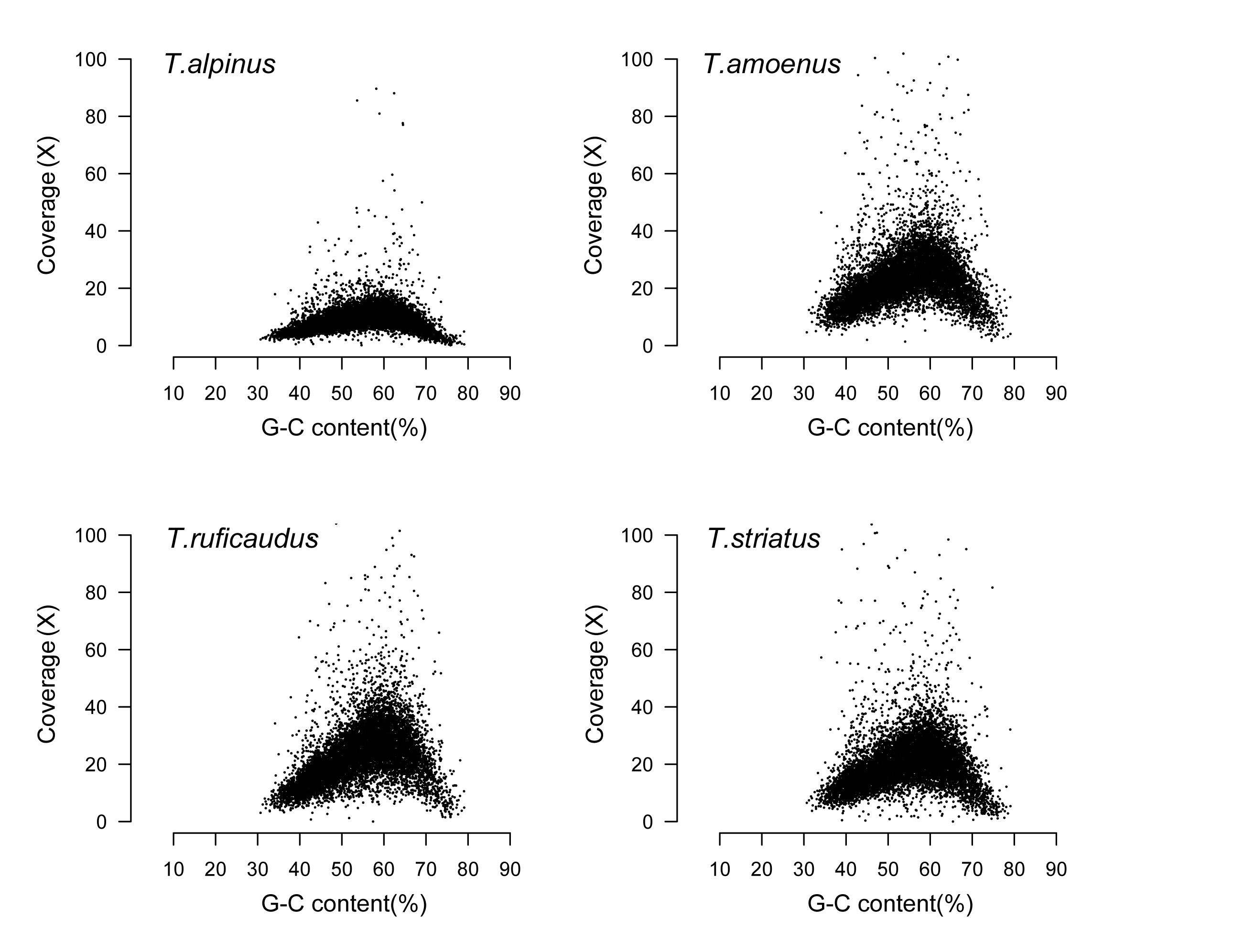
**

**Figure S5**

**
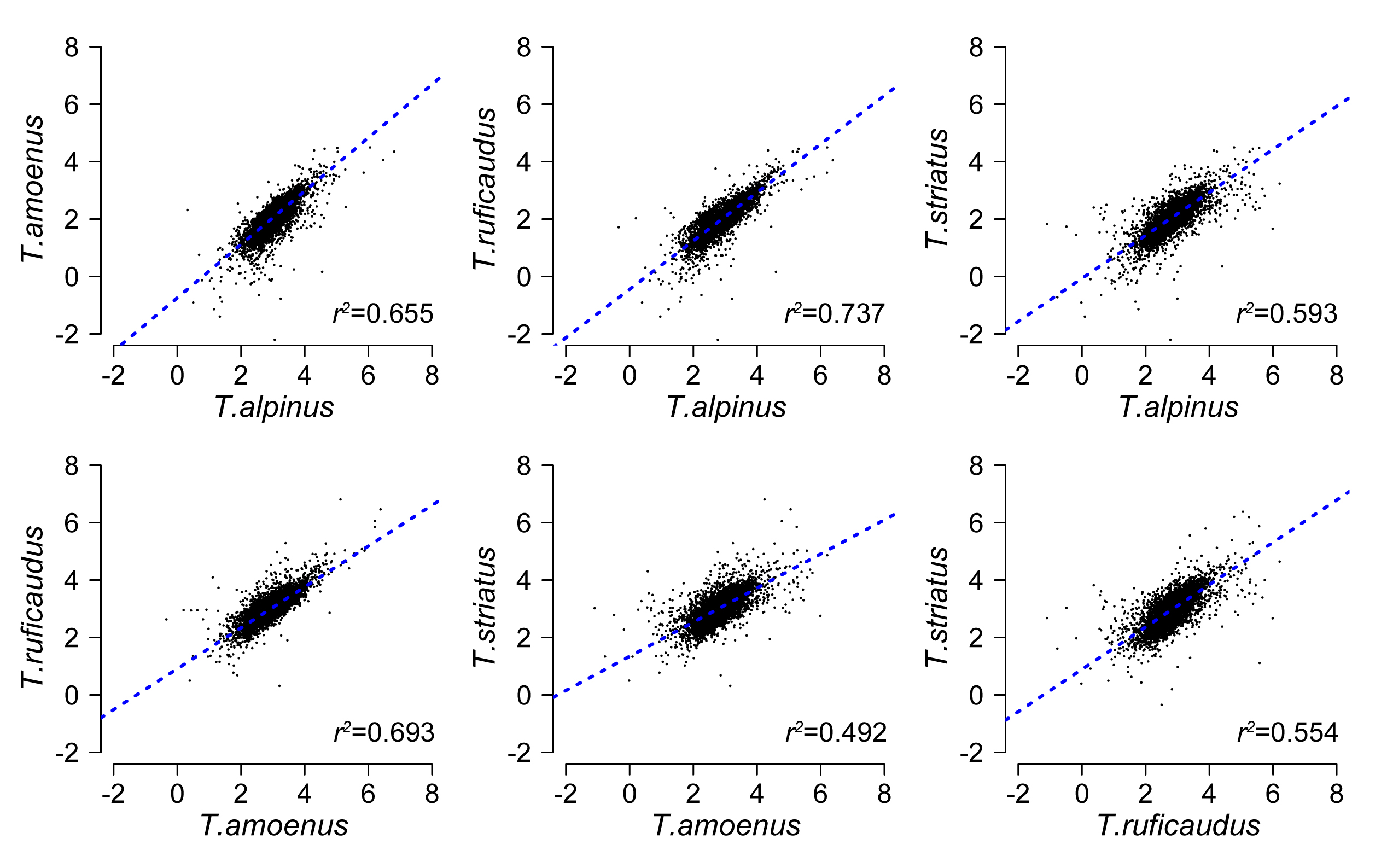
**

**Figure S6**

**
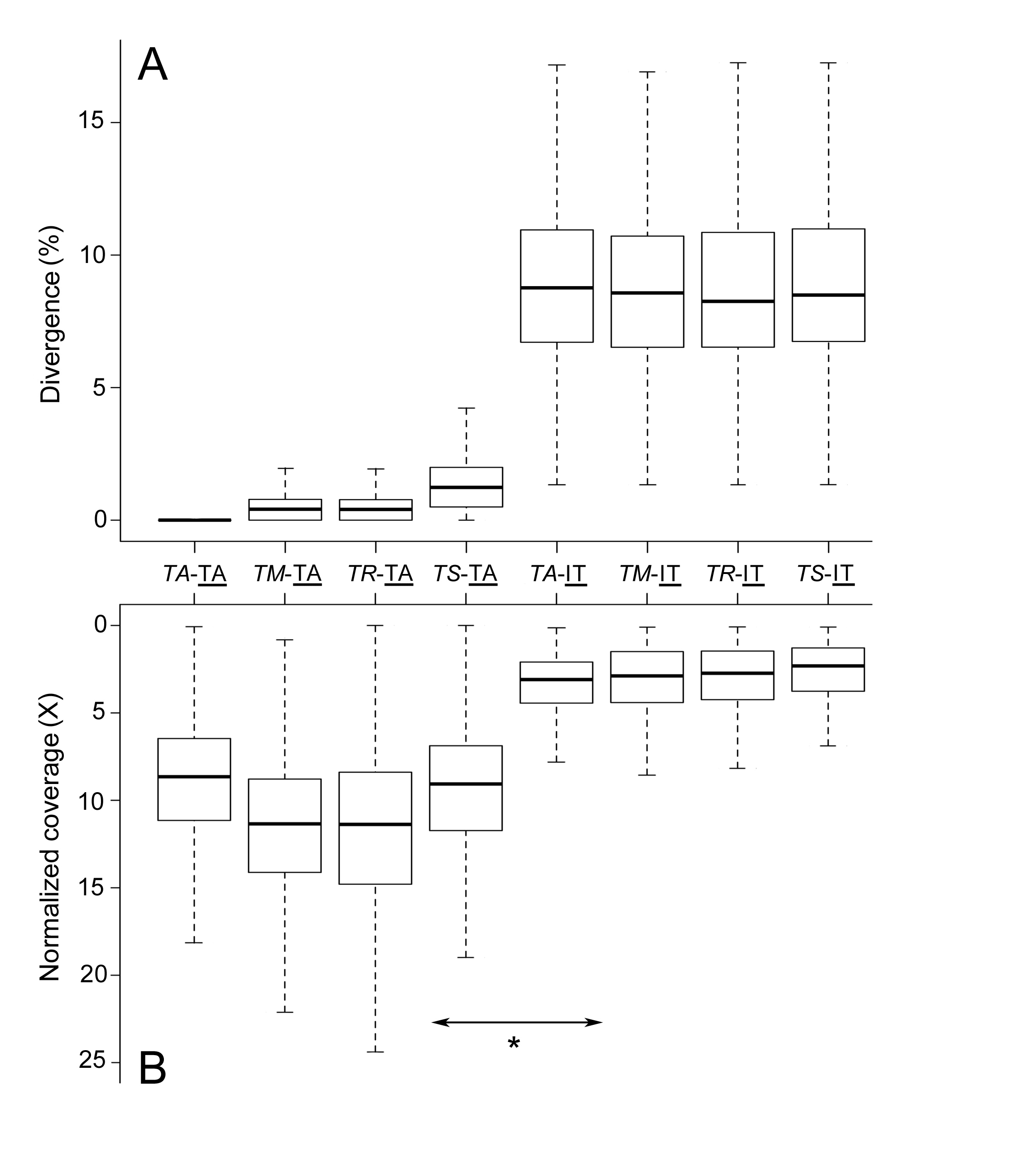
**

**Figure S7**

Supplement: Additional file 3 — Supplementary figures (Figures S1-S7). Figure S1. Correlation between the length of annotated transcripts and the average base coverage. Figure S2. Divergence of orthologous transcripts between Tamias alpinus and Urocitellus beldingi. The sequence divergence between target exons selected in the present study and their orthologous genes in Urocitellus beldingi (red) follows a similar distribution pattern to that of all identified orthologous pairs between the two species (black). Figure S3. Raw assemblies of captured sequences from the four chipmunk species. The raw assemblies generated by ABySS and by SOAPdenovo were combined for the summary. The mean and median lengths, and N50 of raw assemblies generated using various combinations of k-mer and k-cov were similar among species. Black bars indicate standard deviation. Figure S4. Sequence coverage of captured mitochondrial genome from each chipmunk species. The genes/regions of each Tamias mitochondrial contig were re-organized by comparing them to the reference mouse (Mus musculus) mitochondrial genome obtained from NCBI (JQ003190.1). The top horizontal bar depicts gene annotations except for the tRNA genes. A. Tamias alpinus; B. T. amoenus; C. T. ruficaudus; D. T. striatus. Figure S5. Impact of variable GC content of exons on the capture efficiency. Capture efficiency is shown by average base coverage within each target exon. Figure S6. Comparison of performance of exon capture in independent capture experiments. Exon base coverage was log transformed and is shown on the X- and Y-axis. There is strong correlation in capture efficiency between the same targets in different species libraries, captured on separate arrays. Figure S7. Sequence divergence (A) vs. normalized base coverage (B) in divergent and closely related targets. TA: Tamias alpinus; TM: T. amoenus; TR: T. ruficaudus; TS: T. striatus; IT: Ictidomys tridecemlineatus. TA: Target exons of T. alpinus; IT: Target genomic regions of I. tridecemlineatus. Ther [file 1471-2164-13-403-S3.docx]
